# Supplementary figures and images for: Rational design of structure‐based vaccines targeting misfolded alpha‐synuclein conformers of Parkinson's disease and related disorders
Source: Bioeng Transl Med. 2024 Apr 9;9(4):e10665. doi: 10.1002/btm2.10665 (PMC11256163; doi:10.1002/btm2.10665)

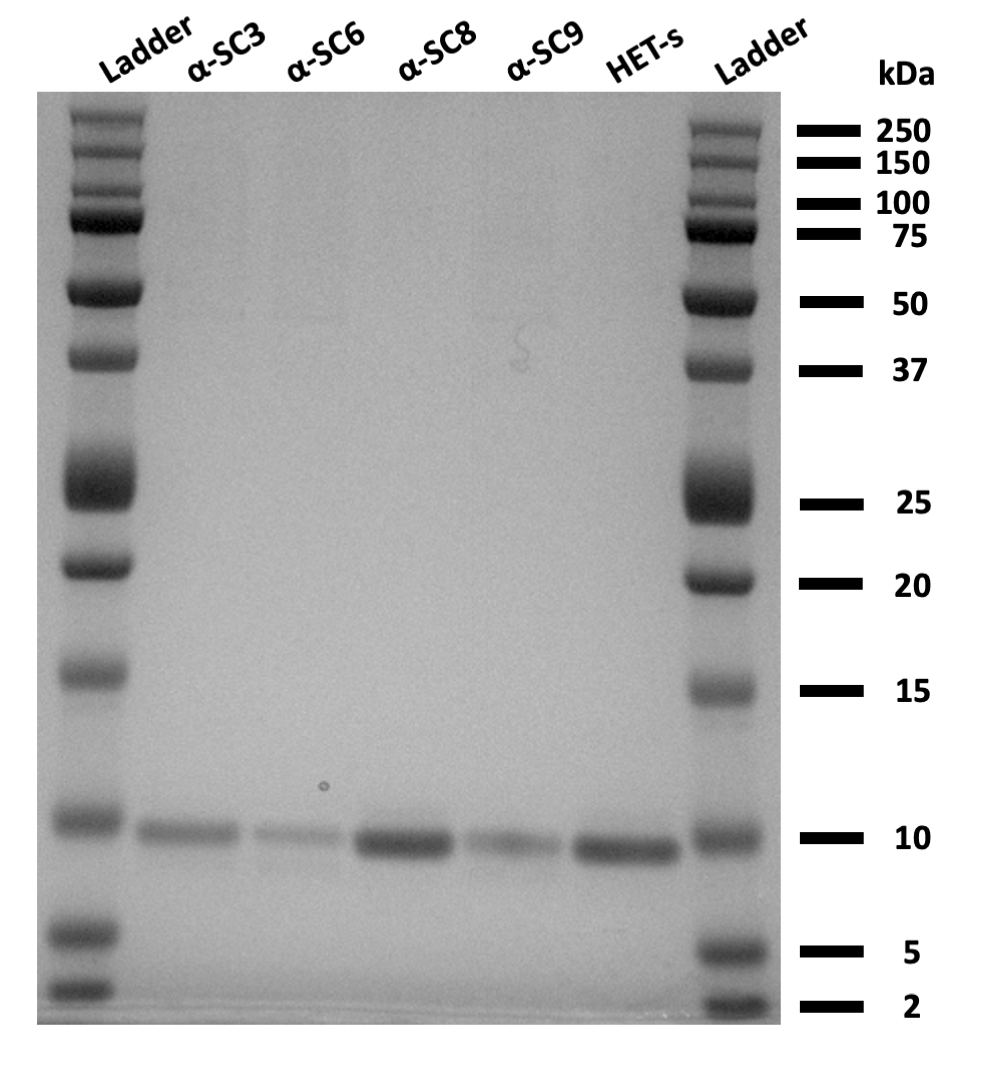

Supplement: Supplementary file 1 — Figure S1. Successful production and purification of alpha‐synuclein‐targeting vaccine candidates for further study. The engineered proteins were optimized for expression in Escherichia coli, purified by affinity chromatography, and desalted. The resulting proteins were resolved on a NuPAGE 12% Bis‐Tris protein gel, and found to have a molecular mass not significantly different from the original scaffold protein. Gel electrophoresis was used as a quality control measure for all batches of antigens produced. [file BTM2-9-e10665-s001.tif]
